# EGR1 controls divergent cellular responses of distinctive nucleus pulposus cell types

**DOI:** 10.1186/s12891-016-0979-x

**Published:** 2016-03-14

**Authors:** Guus G. H. van den Akker, Don A. M. Surtel, Andy Cremers, Martijn F. G. A. Hoes, Marjolein M. Caron, Stephen M. Richardson, Ricardo Rodrigues-Pinto, Lodewijk W. van Rhijn, Judith A. Hoyland, Tim J. M. Welting, Jan Willem Voncken

**Affiliations:** Department of Orthopaedic Surgery, Maastricht University Medical Centre, Maastricht, The Netherlands; Department of Molecular Genetics, Maastricht University Medical Centre, Maastricht, The Netherlands; Centre for Tissue Injury and Repair, Institute of Inflammation and Repair, The University of Manchester, Manchester, UK; NIHR Manchester Musculoskeletal Biomedical Research Unit, Manchester Academic Health Science Centre, Manchester, UK; Current Address: Department of Experimental Rheumatology, Radboud University Medical Center, Nijmegen, The Netherlands; Current Address: Department of Orthopaedics, Centro Hospitalar do Porto - Hospital de Santo António, Porto, Portugal

**Keywords:** Intervertebral disc, Nucleus pulposus, Cell line, EGR1, Specific cell responses, IL-1β, Inflammation, Differentiation

## Abstract

**Background:**

Immediate early genes (IEGs) encode transcription factors which serve as first line response modules to altered conditions and mediate appropriate cell responses. The immediate early response gene EGR1 is involved in physiological adaptation of numerous different cell types. We have previously shown a role for EGR1 in controlling processes supporting chondrogenic differentiation. We recently established a unique set of phenotypically distinct cell lines from the human nucleus pulposus (NP). Extensive characterization showed that these NP cellular subtypes represented progenitor-like cell types and more functionally mature cells.

**Methods:**

To further understanding of cellular heterogeneity in the NP, we analyzed the response of these cell subtypes to anabolic and catabolic factors. Here, we test the hypothesis that physiological responses of distinct NP cell types are mediated by EGR1 and reflect specification of cell function using an RNA interference-based experimental approach.

**Results:**

We show that distinct NP cell types rapidly induce EGR1 exposure to either growth factors or inflammatory cytokines. In addition, we show that mRNA profiles induced in response to anabolic or catabolic conditions are cell type specific: the more mature NP cell type produced a strong and more specialized transcriptional response to IL-1β than the NP progenitor cells and aspects of this response were controlled by EGR1.

**Conclusions:**

Our current findings provide important substantiation of differential functionality among NP cellular subtypes. Additionally, the data shows that early transcriptional programming initiated by EGR1 is essentially restrained by the cells’ epigenome as it was determined during development and differentiation. These studies begin to define functional distinctions among cells of the NP and will ultimately contribute to defining functional phenotypes within the adult intervertebral disc.

**Electronic supplementary material:**

The online version of this article (doi:10.1186/s12891-016-0979-x) contains supplementary material, which is available to authorized users.

## Background

Cell models provide valuable tools to study important aspects of cell and tissue biology, including development, differentiation and physiological adaptation to changes in the cellular environment. Such micro-environmental changes include mitogenic or differentiation stimuli, cell-cell and cell-extracellular matrix (ECM) contacts or exposure to catabolic factors such as inflammatory cytokines. Despite their close resemblance to cells in their native tissue environment, primary cell models often have restricted application due to their limited proliferative capacity, cellular heterogeneity and poor definition of appropriate culturing conditions. Combined, these factors will cause any physiological response in primary isolates to be an average of multiple, potentially different, responses by a variety of cell types. As such immortal cell lines provide powerful tools to explore isolated cell biology under normal and pathological conditions.

Cells of the nucleus pulposus (NP) and annulus fibrosus (AF), the two main tissues that form the structure of the intervertebral disc (IVD), are responsible for IVD homeostasis. The gelatinous NP mediates central absorption of mechanical forces, whereas the surrounding fibrous AF secures the positioning of the NP and connects the IVD to the adjacent endplates and vertebra [1]. Human beings will develop some degree of lower back pain and/or degenerative disc disease (DDD) as they age [2]. DDD is associated with aberrant IVD cell function, reduced cellularity and concomitant loss of proteoglycans and tissue dehydration [3]. Although IVD cells play an important role in DDD, to date, knowledge on the exact nature and involvement of different NP and AF cell types in IVD development and DDD is far from complete. Development of the AF is closely related to definition of somites during embryogenesis, whereas a growing number of reports support the notion that all mature NP cells are derived from notochordal precursor cells [4, 5]. In humans, the presence of cells that express *Brachyury T* (*T*) sets apart a progenitor-like notochordal cell (NC) type. As these NC cells disappear during adolescence, the human IVD differs considerably from the IVD in other species, which appears to retain a low percentage of NC cells [1].

Cellular heterogeneity in the human NP is poorly understood. Accumulating evidence from in vivo and in vitro experimental observations, suggests that the mature NP comprises multiple cellular subtypes [6–10]. Although little is known about these NP cell subpopulations this cellular heterogeneity may reflect cellular differentiation stages. The application of human IVD-derived cell material as a potential source of cell lines has been limited, with few studies reporting immortalized IVD cell models [9, 11]. Despite the restricted availability of immortal IVD cell lines, such models can provide important experimental tools to study essential aspects of IVD biology, ranging from the tracking of developmental origins of the NP and AF by marker gene expression to defining cellular targets in DDD. A detailed understanding of ontogenic, cellular and molecular characteristics of IVD cell populations will be valuable to increase our understanding of the contribution of such cells to DDD, for targeted drug development, drug testing for DDD, and ultimately for the development of cell replacement therapies and IVD regeneration.

We recently set out to generate and characterize immortal human NP and AF clonal cell lines [6, 12]. The main aim of these studies was to determine whether cellular heterogeneity was reflected in the immortalized pool of NP or AF cells. Importantly, the immortalization procedure appeared to have fixed distinctive cellular phenotypes: distinctive NP and AF cellular subtypes were distinguished based on morphological characteristics, on the basis of specific gene and/or protein expression profiles and their response to specific culturing and/or differentiation conditions [6, 12]. Hence, increased insight in composition of the NP cell population may help define their involvement in pivotal aspects of NP development, maintenance and disease.

Immediate early response genes (IEG) comprise a larger family of responder genes that provide an essential link between the micro-environment of a cell and its physiology. IEGs encode transcription factors that govern genomic responses to altered demands, thereby enabling phenotypic plasticity. Despite their well-established involvement in relaying mitogenic and stress responses, relatively little is known about the involvement of IEGs in skeletal development and homeostasis. The *Early Growth Response gene 1* (*EGR1; KROX-24; NGFI-A; TIS8; ZIF-268; ZENK*) has been functionally associated to biological aspects of mesodermal derivatives (adipogenesis and tenogenesis), yet relatively little is known about its function in the IVD [13–15]. We recently established that the IEG EGR1 mediates early chondrogenic responses of ATDC5 cells upon exposure to differentiation conditions in vitro [16]. In addition, EGR1 has been associated with acute inflammatory and regenerative responses [17–22]. Thus EGR1 may fulfill a number of crucial tasks at the cross roads of physiological, inflammatory and regenerative responses in the IVD.

We here outline a study that begins to examine the involvement of EGR1 in cell type specific responses of distinct NP subtypes to anabolic and catabolic stimuli. By ablating EGR1 function using RNA interference, we find that EGR1 mediates functionally distinct responses in these NP cellular subtypes. The impact of these findings on understanding NP cellular heterogeneity is discussed.

## Methods

### Intervertebral disc cells, cell culture and immortalization

The generation of immortal non-degenerate healthy disc cells has been described elsewhere [6]. Immortalized NP cells were cultured in maintenance medium (DMEM-F12/Glutamax (Gibco), 10 % fetal calf serum (FCS; Biowhittaker, cat no DE14-801 F), 1 % penicillin/streptomycin (Gibco), 1 % non-essential amino acids (NEAA; (Gibco)). Cells were maintained by 1:4 splits (1 passage equals 2 population doublings) seeded at a density of 30,000 cells/cm^2^. Unless stated otherwise, representative AF (AF-123) and NP (NP-nR105; NP-R115) clones are utilized for experimentation in this study. Disc material was obtained as surplus material from correction surgery (Medical Ethical Review Committee approval 08-4-021) [6].

### Growth factors

Cells were seeded in monolayer (8000 cells/cm2) in basal medium. At day 2, medium was aspirated and cells were washed twice with PBS. Cells were cultured (3 independent experiments) for 2 days in Differentiation Medium (DMEM/F12 + 1 % Pen/Strep + 1 % NEAA + 1 % ITS + 1 % ascorbate) containing: no growth factor (control), TGFβ3 (10 ng/ml, Life technologies), GDF6 (100 ng/ml, Peprotech), CTGF (100 ng/ml, Peprotech), Il-1β (10 ng/ml, Peprotech) and TNFα (50 ng/ml, Peprotech).

### RNA-interference based EGR1 knock-down

The sense siRNA sequence for human *EGR1* was 5′-ACGACAGCAGUCCCAUUUATT-3′ and the anti-sense sequence was 5′-UAAAUGGGACUGCUGUCGUTT-3′. A scrambled siRNA-duplex was used as control; both sequences were designed using algorithms provided by the vendor (Eurogentec). IVD cell lines were seeded at 20,000 cells/cm^2^ and transfection with siRNAs was performed using ICAfectin 442 (Eurogentec) according to manufacturers’ instructions. Procedures were essentially as described before [16, 23]. Cells were cultured for 16 h following siRNA transfection before stimulations were performed. *EGR1* siRNA concentration was optimized at 30 nM in parallel in murine and human cell lines (Additional file 1: Figure S1A, B). Sustained *EGR1* knock-down at 16 + 48 h, was verified in ITS-treated NP cells (Additional file 1: Figure S1C).

### RNA isolation and quantitative real time PCR

For RNA isolation, cells were disrupted in Trizol (Invitrogen). RNA isolation, RNA quantification (UV)-spectrometry (Nanodrop, Thermo Scientific), and cDNA synthesis were performed as described before [20]. Real-time quantitative PCR (RT-qPCR) was performed using Mesagreen qPCR master mix plus for SYBR® Green (Eurogentec). Validated primer sets used are depicted in Table 1. An Applied Biosystems ABI PRISM 7700 Sequence Detection System was used for amplification: initial denaturation 95 °C for 10 min, followed by 40 cycles of DNA amplification. Data were analyzed using the standard curve method and normalized to *Cyclophillin B* (Cyclo).

**Table 1:** rtPCR primer sets for gene expression measurements

Symbol	Forward primer	Reverse primer
ADAMTS4	GGTCATGTCTTCAACATGCTCC	AGGATCCACATGAGCCATCAC
COL1A1	TGGAGAGTACTGGATTGACCCC	TGCAGAAGACTTTGATGGCATC
COL2A1	TGGGTGTTCTATTTATTTATTGTCTTCCT	GCGTTGGACTCACACCAGTTAGT
COX2	ACCAACATGATGTTTGCATTCTTT	GGTCCCCGCTTAAGATCTGTCT
EGR1	TGACCGCAGAGTCTTTTCCT	TGGGTTGGTCATGCTCACTA
KRT19	GCAGTCACAGCTGAGCATGAA	TCCGTTTCTGCCAGTGTGTCT
MMP3	TGATGAACAATGGACAAAGGATACA	TTTCATGAGCAGCAACGAGAA
SOX9	AGTACCCGCACCTGCACAAC	CGCTTCTCGCTCTCGTTCAG
T	CCACCTGCAAATCCTCATCCT	TTGGAGAATTGTTCCGATGAG
TNFα	TCAATCGGCCCGACTATCTC	CAGGGCAATGATCCCAAAGT
VCAN	TCCCTCACTGTGGTCAAG	GTGTGTACCTGCTGGTTG
βACT	CCTGGCACCCAGCACAAT	GCCGATCCACACGGAGTACT
CYCLO	CCTGCTTCCACCGGATCAT	CGTTGTGGCGCGTAAAGTC
EIF2B1	TGTCAGGTAAGAAAATGGCCAAA	TGTAGCCGACAGCAGCATCT
MRPL19	CGCCGAAACCGGTCATC	TCCCCTTCGAGGAATGAATTC

### Protein extraction and immunoblotting

Protein extraction and immunoblotting were performed and analyzed as described previously with minor adjustments [21]. For extraction, cells were lysed in RIPA buffer (50 mM Tris pH 8.0, 150 mM NaCl, 0.1 % SDS, 5 mM EDTA, 0.5 % w/v Sodium Deoxycholate, and 1 % NP-40) supplemented with protease and phosphatase inhibitor (Roche). Lysates were sonicated on ice using the Soniprep 150 MSE at amplitude 10 for 14 cycles (1 s on/1 s off). Insoluble material was removed by centrifugation (10 min, 16000 × g, 4 °C). Protein concentration was determined using a BCA protein assay kit (Pierce/Thermo Fisher Scientific). Proteins samples were separated by SDS-PAGE and immobilized on Nitrocellulose membranes. Membranes were blocked (1 h, 5 % non-fat dry milk powder (Campina), ambient temp), and incubated with primary antibodies (overnight, 4 °C). Antisera: mouse monoclonal EGR1 (Abcam 55160), rabbit polyclonal β-Actin (C4, 691001; MP Biomedicals), Secondary antisera: rat anti-mouse (P0260; DAKO, Glostrup, Denmark) and donkey anti-rabbit (711035–152; Jackson Lab, Bar Harbor, ME, USA). Signals were detected using enhanced chemoluminescence (Pierce).

### Immunohistochemistry

Research involving human material was performed in accordance with the Declaration of Helsinki. Human IVD tissue for histology was obtained from a 63 years old deceased (non-heart beating) donor. Approval for all experimental sections of the current study and informed consent for publication of patient details and accompanying images in this manuscript was obtained as an integral part of the MUMC-Medical Ethical Review Committee (METC approval 08-4-021; July 11, 2012); the approval is held by the authors (LWvR) and is available for review by the Editor-in-Chief. Two lumbar IVDs L1/2 (without overt signs of degeneration; healthy) and L4/L5 (clearly degenerated) were dissected by an orthopedic surgeon. Tissue was isolated, fixed in formalin and decalcified in 0.5 M EDTA pH 7.8 for two weeks. EDTA was refreshed every two days. Tissues were dehydrated and embedded in paraffin and five micrometer section were cut and positioned on Superfrost Plus slides. For immunohistochemical analysis, sections were deparaffinized and rehydrated using standard protocols. Antigen retrieval was performed in hot citrate buffer (1.8 mM citric acid and 8.2 mM tri-sodium citrate), endogenous peroxidase activity was inactivated by Peroxidase-Blocking solution (Dako, REAL) and samples were blocked with 10 % normal sheep serum. Rabbit polyclonal anti-Egr-1 (Santa Cruz Biotechnology) was incubated overnight at 4 °C in 1:200 concentration (primary antibody was not used for negative control sections). Unbound antibodies were removed by washing in PBS with 0.1 % Tween. Bound primary antibodies were detected with HRP-labelled anti-rabbit secondary antibodies (EnVision + System-HRP labelled Polymers; Dako) by incubation for 30 min at room temperature. For detection, DAB chromogen substrate (Dako) was used. Stained sections were counterstained with Mayor’s Hematoxylin (Dako) and subsequently dehydrated and mounted in Histomount (Thermo Shandon) for microscopic analysis.

### Statistics

Statistical significance (*p* < 0.05) was determined by two-tailed Student’s *t*-test. To test for normal distribution of input data, D’Agostino–Pearson omnibus normality tests were performed. All quantitative data sets presented passed the normality tests. Gene expression analyses show mean and standard deviation (as indicated); indicated *p*-values in figures represent the comparison between AF and NP for all donors combined or between clonal subtypes (NP-R vs NP-nR).

## Results

### Differentiation induces EGR1 in IVD cells

**Fig. 1:**
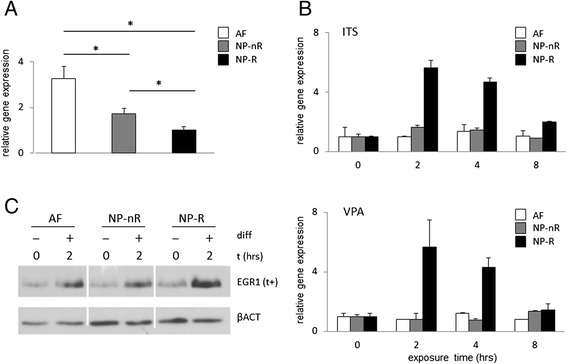
Induction of EGR1 expression in IVD cell lines. **a** Basal expression of *EGR1* mRNA in representative clones (AF-123, NP-nR 105 and NP-R 115). Gene expression was normalized to *Cyclophillin B* and is presented relative to the NP-R clone. **b** Insulin, Transferrin and selenite (ITS; 10 μg/ml insulin, 10 μg/ml transferrin and 3 × 10^−8^M sodium selenite) and Valproic acid (0.3 mM) were used to stimulate IVD cell lines for 0, 2, 4 and 8 h. Gene expression of *EGR1* was normalized to *Cyclophillin B* and is presented relative to the t = 0 time point. Bars represent a biological triplicate. **c** Protein expression of EGR1 under basal conditions and in response to stimulation with chondrogenic media for two hours. AF, NP-nR and NP-R samples were run on the same gel to allow direct comparison. The indication (t+) points to a long apposition time. Βeta Actin (βACT) was used as loading control; asterisks indicate significantly different expression level; *p* < 0.05

A detailed description of the NP cellular subtypes used in the current study is published elsewhere [6]. We have previously established that chondrogenic ATDC5 cells strongly induce EGR1 mRNA and protein expression in response to chondrogenic differentiation stimuli [16]. To investigate whether IVD cells activate EGR1 expression in response to changes in their environment, we initially subjected immortal AF and NP cell lines to previously defined chondrogenic differentiation conditions [16]. Typically, IEG expression in most cell types is low or absent under basal conditions and, upon exposure to differentiation conditions, increases within hours at the mRNA and protein levels. Basal *EGR1* mRNA expression was approximately 2–4 fold higher in immortal AF cells than in two phenotypically distinct NP cell types of which the NP-R cell type showed the lowest *EGR1* mRNA levels (Fig. 1a). Exposure of these IVD cell types to chondrogenic differentiation conditions resulted in a robust *EGR1* mRNA induction (±6 fold) at 2 h post-induction in NP-R cells (Fig. 1b; upper panel); the maximum response of NP-nR cells did not reach twofold, whereas AF cells did not show any induction of EGR1 at the 2 h time point (Fig. 1b, upper panel). Valproic acid (VPA), a known inducer of IEGs [24, 25], was used in a parallel experiment as an intended positive control. VPA exposure resulted in a pronounced upregulation of *EGR1* mRNA, although, surprisingly, exclusively in NP-R cells; as with ITS, no *EGR1* mRNA induction was detected in NP-nR and AF cells (Fig. 1b, lower panel). The twofold increase of EGR1 mRNA at 8 h post-induction in NP-nR cells was significant, but did not qualify as an IEG response.

We next examined EGR1 induction at the protein level. EGR1 protein was detectable in vitro at relatively low levels in all three IVD cell types, and was rapidly induced within 2 h after stimulation with chondrogenic differentiation medium in all cells (Fig. 1c). Despite the poor correlation of basal mRNA and protein levels in these respective cell lines under control conditions, mRNA (*cf.* Fig. 1b) and protein levels upon stimulation with ITS were consistently highest in the NP-R (responder) clone. This data suggests that upregulation of EGR1 protein in IVD cells is accompanied by an induction of *EGR1* mRNA preferentially in NP-R cells. Given that AF cells did not respond, further analysis did not include these cells.

### Distinct physiological responses in specialized NP cellular subpopulations

**Fig. 2:**
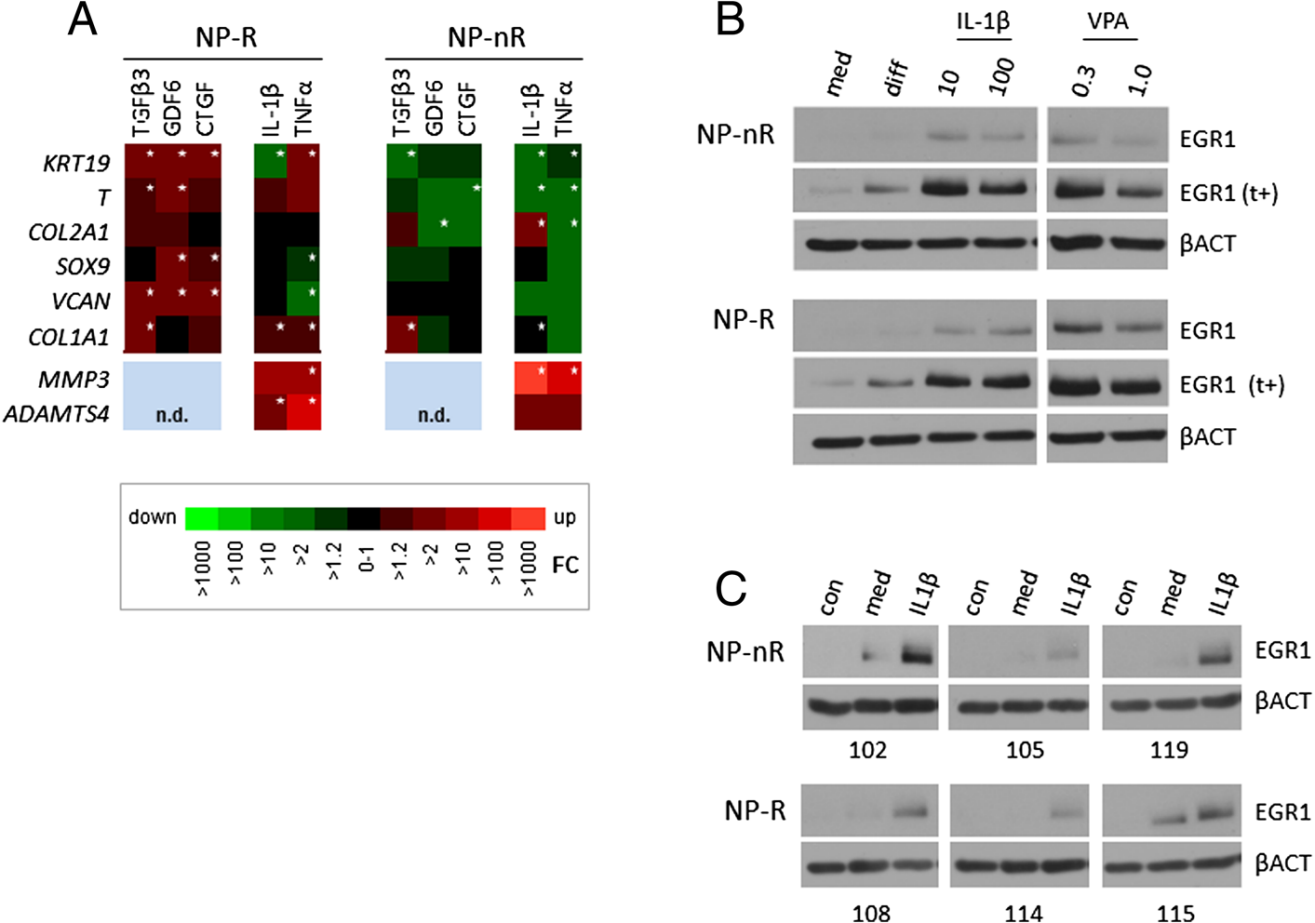
Cytokines induce catabolic response in the mature NP subclones. **a** Heatmap representation of gene expression changes in distinct NP subclones. NP cell clones NP-nR 105 and NP-R 124 were stimulated for two days with anabolic factors TGFβ3 (10 ng/ml), GDF6 (100 ng/ml), CTGF (100 ng/ml) or catabolic factors IL-1β (10 ng/ml) or TNFα (50 ng/ml). mRNA levels for the following genes was determined: *KRT19, T, COL2A1, SOX9, VCAN, COL1A1, MMP3* and *ADAMTS4*. Gene expression was normalized to the average of EIF2B1 and MRPL19 expressions. Fold induction was calculated by comparison to untreated cells at day 2 and is represented in a heatmap; *n.d.*: not determined. Asterisks indicate significantly different expression level; *p* < 0.05. **b** Protein expression of EGR1 in response to maintenance medium (med), differentiation medium (diff), IL-1β (10 and 100 ng/ml) or Valproic acid (0.3 and 1 mM). NP-nR 105 and NP-R 115 samples were run on the same gel to allow direct comparison. The indication (t+) points to relatively long apposition times. Β Actin was used as loading control. **c** Three NP-nR and three NP-R clones were either left undisturbed (con), exposed to a change of maintenance medium (med) or medium supplemented with IL-1β (10 ng/ml) for two hours. Non-stimulated cells at time point t = 0 were used as control (con). Βeta Actin (βACT) was used as loading control

In a recent report, we speculated that the distinct immortal NP subtypes differ in differentiation/maturation status [6]. This previous data suggested that NP-R cells represent a more progenitor–like phenotype and that NP-nR are more mature, differentiated cells, as they do not produce the robust *SOX9* response that NP-R cells show upon exposure to differentiation conditions. In line with this idea, we have previously shown that chondrogenic differentiation of ATDC5 cells is critically dependent on EGR1 [16]. Based on these findings we speculated that the distinct NP cellular subtypes may be differentially sensitive to specific physiological stimuli. In line with the above observations, we predicted that NP-R would respond more robustly to differentiation stimuli, whereas NP-nR may be more sensitive to degenerative stimuli. To this end NP-nR and NP-R cells were subjected to anabolic (*i.e.* growth factors) or catabolic conditions (*i.e.* inflammatory cytokines), representing potential regenerative or degenerative stimuli, respectively. Transcriptional regulation of a number of marker genes was measured in response to differentiation conditions, among which: *KRT19*, *T* (Keratin 19 and Brachyury T), *SOX9* (SRY box-9), *COL2A1*, *COL1A1* (type 2 and 1 alpha Collagens) and *VCAN* (Versican), from here on referred to as *IVD marker genes*. In addition, expression of a number of catabolic markers, including *MMP3* (Matrix Metalloproteinase 3) and *ADAMTS4* (A Disintegrin and Metalloproteinase with Thrombospondin motifs 4,5) was determined. The biological responses to anabolic or catabolic stimuli showed remarkable differences between NP-R and nR subtypes: overall anabolic conditions (growth factor stimulation) induced expression of the IVD markers *SOX9*, *COL2A1*, *COL1A1*, *VCAN*, *KRT19* and *T* in NP-R cells (Fig. 2a). Conversely, exposure to different growth factors produced an overall transcriptionally repressive response of IVD marker gene expression in NP-nR cells. This overall effect on transcription of these IVD markers was clearly less unidirectional in NP-R cells. The only conspicuous exception was TGFβ3-induced *COL1A1* and *COL2A1* induction in the NP-nR subtype. NP-nR cells showed a massive induction of *MMP3* in response to IL-1β or TNFα (±1000 fold or 100 fold, respectively), whereas this response was less pronounced in the NP-R clones (±10–100 fold less). The *ADAMTS4* marker was expressed at similar levels in both NP subtypes in response to IL-1β and was slightly up in the TNFα NP-R cells. This data suggests that, phenotypically distinct NP-R and NP-nR cells also differ in terms of responses to anabolic and catabolic conditions.

### EGR1-dependent responses in NP cellular subtypes

EGR1 has been reported to mediate transcriptional responses to inflammatory cytokines including IL-β [18, 19, 26, 27]. We therefore investigated the EGR1-dependence of the catabolic responses to IL-1β, as this cytokine induced clear differences in gene response profiles between NP-R and NP-nR cells. We first examined the EGR1 protein induction in response to IL-1β stimulation in more detail. NP-R and NP-nR cells were exposed to IL-1β for 2 h, and EGR1-induction was compared in whole cell extracts; maintenance medium, differentiation medium or VPA exposed cell extracts (2 h) were taken as reference conditions. As before, NP cells induced EGR1 protein in response to differentiation conditions. Both NP-nR and NP-R cells produced an obvious EGR1 induction in response to either 10 or 100 ng/mL IL-1β (Fig. 2b). We evaluated IL-1β-induced EGR1 response in multiple independent NP-nR and NP-R clones: all clones showed a robust induction of EGR1 protein upon 2 h of IL-1β treatment (Fig. 2c). Combined, this data shows that both anabolic and catabolic conditions induce an immediate early response in IVD cell types. Combined, our previous observations [6] and the data presented thus far, shows that although EGR1 is induced in both NP cell subtypes by differentiation factors and inflammatory cytokines, their transcriptomic responses are dissimilar.

**Fig. 3:**
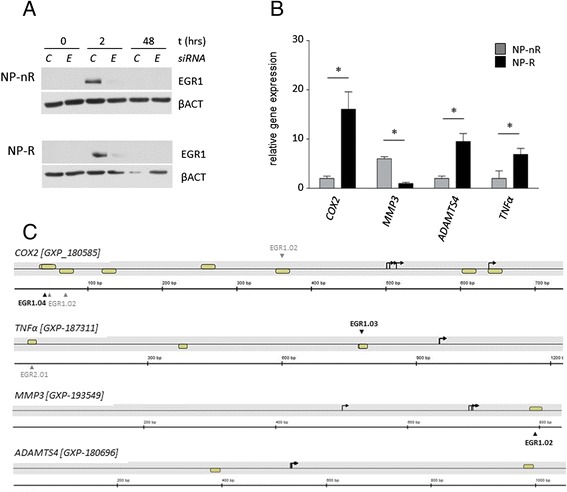
Confirmation loss of EGR1 function. **a** NP-nR and NP-R clones were transfected with 30 nM siRNAs targeting *EGR1* (E) or control (C) siRNA. Sixteen hours after transfection cells were harvested (time point t = 0) and stimulated with IL-1β for 2 and 48 h. Immunoblotting for EGR1 reveals efficient knock-down of *EGR1* at two hours post stimulation in both cell lines. Βeta Actin (βACT) was used as loading control. **b** Basal expression of *COX2*, *TNFα*, *MMP3*, and *ADAMTS4* genes implicated in disc degeneration at t0. Gene expression was normalized to *Cyclophillin B*; asterisks indicate significantly different expression level; *p* < 0.05. **c** *In silico* representation of the promotor region of genes implicated in disc degeneration (*Genomatix software*; http://www.genomatix.de). Putative transcription factor (TF) binding sites for EGR family members are depicted by yellow boxes; high-probability EGR binding sites are indicated by black/bold print arrow heads, low-probability binding sites by light grey/normal print. Black arrows indicate the transcription start site

To further investigate a potential involvement of EGR1 in the transcriptomic response to inflammatory cytokines like IL-1β, an siRNA duplex was designed with the aim to reduce cellular EGR1 mRNA and protein levels; a robust knock-down was obtained at *EGR1* siRNA concentrations of 30 and 100 nM (Additional file 1: Figure S1A), which was specific for *EGR1* as it had no effect on *EGR2* and *EGR3* mRNA levels (Additional file 1: Figure S1B). NP cells were transiently transfected with either 30 nM siRNA targeting human EGR1 (*siEGR1*) or a scrambled control siRNA (*siCTRL*). Cells were treated with IL-1β 16 h following transfection, and samples were taken for expression analysis at 2 and 48 h post-treatment. To verify knock-down throughout the duration of the 2 day induction experiment, EGR1 protein levels were initially compared in *siCTRL* or *siEGR1* transfected cells at 0, 2 and 48 h post-ITS treatment; this analysis showed a robust and sustained reduction of EGR1 protein at 2 and 48 h post-treatment (Additional file 1: Figure S1C). Consistent with the transient nature of IEG induction, the EGR1 protein level was markedly increased at 2 h after IL-1β exposure and had returned to starting (t = 0) levels at 48 h (Fig. 3a). Transfection of NP-R or NP-nR cells with the *siEGR1* successfully diminished EGR1 induction at the protein level 2 h into IL-1β treatment, to nearly undetectable levels (Fig. 3a). At 48 h of IL-1β treatment, EGR1 was neither detectable in the *siCTRL* nor in the *siEGR1*-transfected cells. Thus, the siRNA duplex successfully prevented ERG1 induction in response to IL-1β in both NP-R and NP-nR cells. Moreover, the absence of EGR1 protein at 48 h in the *siEGR1*-condition suggested that the siRNA-mediated targeting strategy did not delay EGR1-induction.

**Fig. 4:**
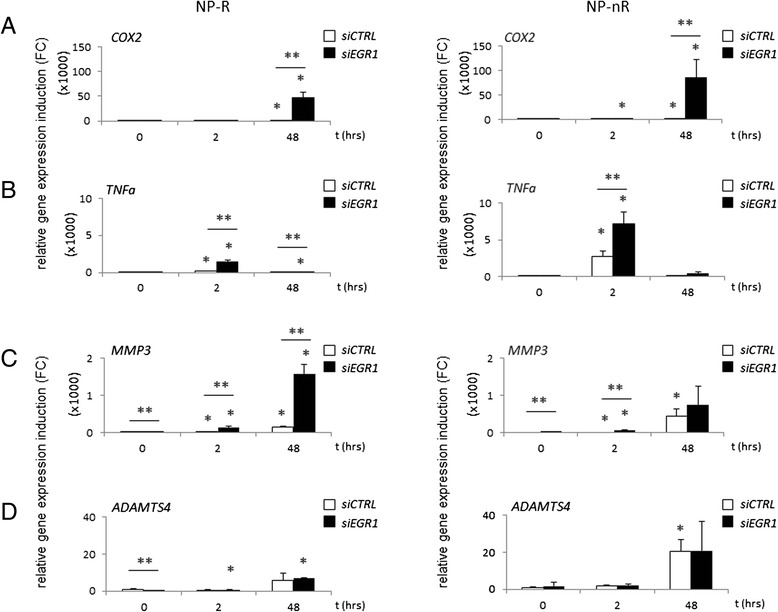
EGR1 controls inflammatory responses in IVD cell clones. Induction of *COX2* (**a**) *TNFα* (**b**) *MMP3* (**c**) and *ADAMTS4* (**d**) expression measured by qPCR. Sixteen hours after siRNA transfection cells were harvested (time point t = 0) and stimulated with IL-1β for 2 and 48 h. Induced mRNA levels are expressed relative (FC) to *siCTRL*, t = 0, for each cell type. Double asterisks (**) indicate significantly different expression levels between *siCTRL* and *siEGR1* measurements at the indicated time points (*p* < 0.05); single asterisks (*) indicate significance (*p* < 0.05) compared to time point t = 0 of that series (*siCTRL* or *siEGR1*)

We then examined expression of a number of genes which are known to be induced by inflammatory cytokines and for which biological relevance in intervertebral disc degeneration had been shown before [28]. These genes included: *COX2, TNFα, MMP3* and *ADAMTS4*. Basal expression measurements in NP-R and NP-nR clones revealed significant differences for all genes: NP-nR cells showed higher basal expression levels for most markers, *MMP3* excepted (Fig. 3b)*.* We have previously shown that chondrogenesis is dependent on intrinsic NF-κB signaling also used by inflammatory cells [23]. In addition, we showed a direct involvement of COX-2 in in vitro and in vivo models for chondrogenesis [29]. We next asked whether expression of *COX2, TNFα, MMP3* and/or *ADAMTS4* was regulated in response to IL-1β, and whether their regulation involved EGR1; the latter was tested by comparing cells expression siRNA against *EGR1* mRNA (*siEGR1*) to cells expressing *siCTRL* (control). The absence or presence of potential EGR family-consensus binding sites in the promoters of the corresponding genes was assessed via www.genomatix.de (Fig. 3c). *COX-2*, *TNFα* and *MMP3* carried potential EGR1 binding sites, whereas *ADAMTS4* did not. *COX-2* was neither induced at t = 2 nor t = 48 h in NP cells, independent of subtype (Fig. 4a). This continued repression of *COX-2* expression appeared to be dependent on EGR1 throughout our experimental setting, as *COX-2* was massively induced at 48 h of IL-1β treatment in NP-nR cells (±60.000 fold) and in NP-R cells (±40.000 fold) that lack EGR1.

NP-nR cells initiated a significantly higher *TNFα* transcription (±3000–4000 fold *versus* <100 fold in NP-R cells) in response to IL-1β (Fig. 4b). As for *COX2, TNFα* expression was negatively controlled by EGR1, as *EGR1* knock-down released *TNFα* mRNA synthesis in both NP cell types at 2 h of IL-1β treatment, this difference was significant from that of *siCTRL* cells. *TNFα* induction in *siEGR1* NP-R cells did not approach the high levels observed in NP-nR cells (±7000 fold versus ±1200 fold). *TNFα* expression appeared to be tightly controlled by EGR1 in the early response phase, as expression ceased at 48 h and appeared no longer dependent on EGR1. Basal *MMP3* gene expression was increased in NP cells transfected with *EGR1* siRNA (Fig. 4c). IL-1β treatment significantly induced *MMP3* expression at 2 h in both cell types. In analogy to the earlier observed more robust induction of *MMP3* in NP-nR *versus* NP-R cells (*cf.* Fig. 2a), we found that *MMP3* followed a similar trend in this experimental setting: IL-1β induced *MMP3* mRNA at higher levels in NP-nR *siCTRL* cells than in NP-R cells at 48 h. Relevantly, in the absence of EGR1 *MMP3* expression was strongly induced at 48 h in NP-R clones. Thus, EGR1 is required to repress inflammatory *MMP3* expression stimulation in NP-R clones. Finally, although IL-1β stimulation induced *ADAMTS4* expression at 48 h in both cell-types, expression levels at 48 h were significantly higher in NP-nR clones, compared to NP-R clones (Fig. 4d). The absence of any effect of EGR1-knock-down on *ADAMTS4* mRNA levels in response to IL-1β, suggests that ERG1 does not directly regulate *ADAMTS4* expression levels and is congruent with the absence of EGR1 binding sites in its promoter.

In summary, the data presented here corroborate the earlier observed differences in gene expression between NP-R and NP-nR clones in response to catabolic stimuli. EGR1 appeared to predominantly act repressive on IL-1β-induced gene expression. For *COX2* and *MMP3,* this was a late effect, suggesting the involvement of additional, secondary transcriptional factor and/or signaling networks in succession to the initial transient EGR1 response, whereas *TNFα* induction was repressed at an early response time point, specifically in NP-R.

**Fig. 5:**
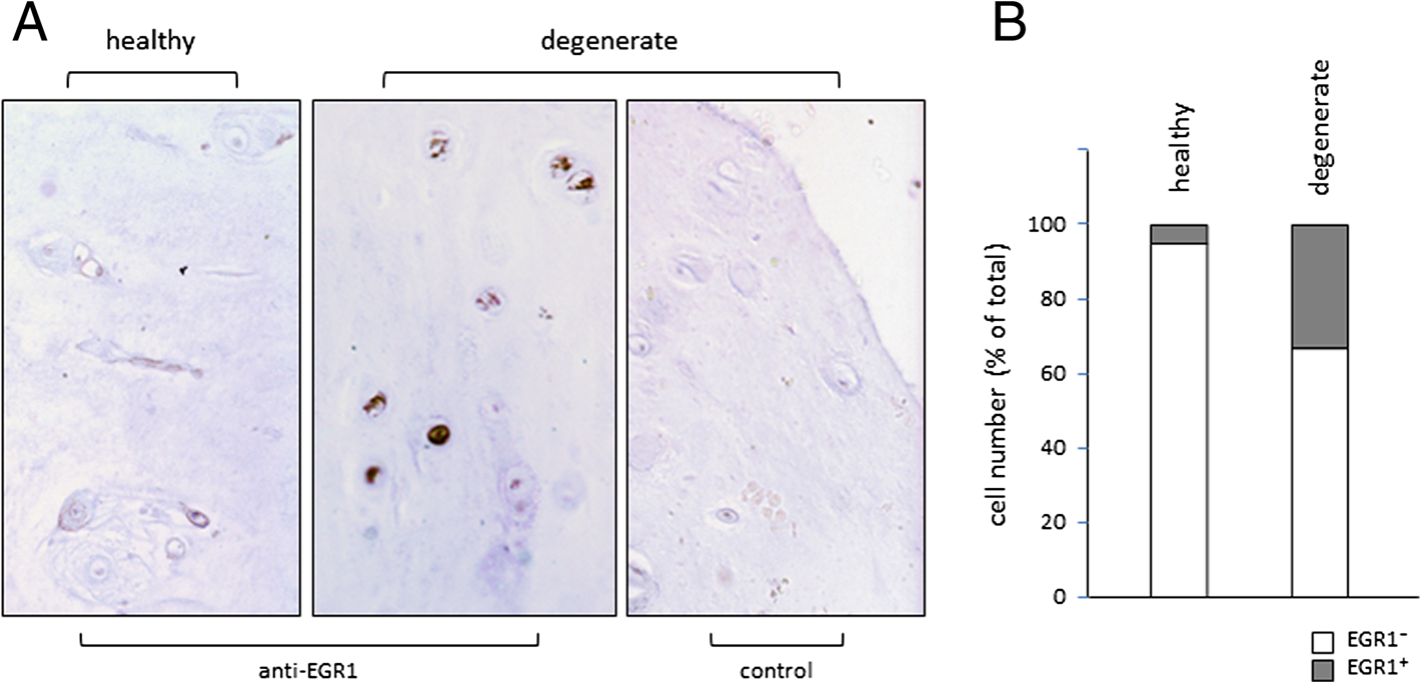
EGR1 expression is increased in degenerate NP cells. **a** Immunodetection of EGR1-positive cells in healthy control NP tissue (L1/L2 IVD of a 63 years old donor; left panel) and in degenerate NP tissue (L4/L5 IVD of the same donor middle panel); right panel: staining control on degenerate NP section: primary antiserum detecting EGR1 was left out. **b** Quantification of EGR+ cell numbers in healthy or degenerate NP tissue. Cell numbers are depicted as percentage of total cell number scored (total cell counts: 121 for healthy tissue and 218 for degenerate tissue)

Thus far the above data show that differentiated/mature NP-nR cells are more responsive to catabolic stimuli than the relatively undifferentiated/progenitor-like NP-R cells, and that aspects of both early and maintained gene expression in response to IL-1β are controlled by EGR1. Based on these findings, we speculated that NP cells expressing EGR1 should be detectable in histological sections of degenerate human intervertebral disc material. Indeed, compared to healthy control section, in which approximately ±5 % of cells in the NP showed EGR1 expression, one in three NP cells in degenerate IVD tissue displayed EGR1-positivity (Fig. 5a, b). Taken together, the data presented support the idea that EGR1 is an important biological response factor in NP cells, and that EGR1 expression is increased under degenerate conditions in the human NP.

## Discussion

We here examined a potential role for the immediate early response gene *EGR1* in various physiological responses of phenotypically distinct immortalized NP cell types. Our combined data shows that the immortalized NP cellular subtypes respond differentially to anabolic and catabolic stimuli. We also provide evidence that the immediate early response gene product EGR1 is differentially involved in cell type-specific responses to IL-1β in immortalized NP-cellular subtypes in vitro. The data presented in this paper thus supports our previous findings that the NP harbors multiple functionally specialized cell types [6].

### Distinct biological functions for NP cell types

Despite the realization that cells in the NP are crucially involved in IVD homeostasis and pathology, little is known about the nature and functionality of such NP cell subpopulations. Several novel lines of evidence support the notion that the NP contains functionally distinct cell types. Recent studies have demonstrated that the NP harbors immature cells with somatic stem cell-like properties [8–10]. We previously reported on a unique experimental immortalization approach to address the issue of NP cellular heterogeneity [6]. We were able to show that multiple phenotypically distinct NP cell types could be immortalized and clonally expanded. The fact that these distinct cellular subpopulations could be isolated from independent donors provided robust and crucial confirmation for the heterogeneous nature of the human NP cell population. Cellular heterogeneity in the NP may reflect different stages of proliferation, differentiation and maturation. Our data thus far suggested that NP-R (responder) clones represented a more immature cell type that harbors an intrinsic capacity to respond to chondrogenic differentiation stimuli. In addition this subpopulation spontaneously formed spheroid structures in (pseudo) 3D cultures [6]. NP-nR (non-responder clones), in contrast, did not induce predicted key developmental control (e.g. *SOX9*) or lineage marker genes (e.g. *CA12*, *FOXF1*, *COMP*) when exposed to chondrogenic conditions and its relatively high membrane-associated CD24 expression suggested that these specific characteristics reflected an ontogenically more advanced stage. To begin to understand the potential biological relevance of these specific NP cell phenotypes, we compared anabolic and catabolic responses of NP-R and NP-nR cells. Our current data show that whereas the NP-R cell response to growth and differentiation factors is substantially more robust than that of NP-nR cells, NP-nR cells, in contrast, appeared to produce a more specialized catabolic response than NP-R cells. Although both cell types display EGR1 induction upon treatment with inflammatory cytokines, the differential transcriptomic response argues that, although the NFκB signaling network in both cell types is operational, the NP-nR may possess a more mature downstream signaling network enabling an EGR1-dependent catabolic response. One striking example thereof is the substantially higher IL-1β-induced *MMP3* mRNA level in NP-nR cells compared to NP-R cells. These findings may be relevant in the context of IVD degeneration: indeed MMPs are involved in DDD [30]. Based on these results it is tempting to speculate that different IVD cell types may have differential contributions to IVD homeostasis and pathology: specific cells maintain homeostasis and harbor potential regenerative capacity, whereas functionally distinct cells may have acquired the ability to respond to inflammatory conditions. CD24-positive NP-nR cells may be sufficiently mature/specialized to partake in catabolic responses to tissue damage, whereas this response would not be beneficial for precursor NP-R cells. Conversely, relatively immature cells would aid in tissue repair, most likely triggered by and/or in response to signals generated by mature cells. In this respect it is interesting to note that IL-1β and TNFα induced a more anabolic profile in the less mature NP-R cells compared to the NP-nR cells (*cf.* Fig. 2a).

### EGR1 regulates NP cell type-specific responses

Understanding the specific immediate early responses of distinct cell types provides insight into genetic and epigenetic wiring of a given cell system. Immediate early response genes (IEG) mediate signaling between the cellular micro-environment and the nucleus. The IEG response is typically transient and serves to control secondary transcriptional responses by acting as facilitators or repressors of gene expression, in conjunction with other transcriptional regulatory factors [31–36]; this enables cells to mount a quick and physiologically relevant response to changed conditions. Such responses may be anabolic, e.g. during differentiation and development or tissue repair, or catabolic, e.g. during inflammation. We reported before that EGR1 fulfills a crucial early role in preparing ATDC5 cells for replicative amplification and transcriptome remodeling that accompany chondrogenic differentiation [16]. Here we provide evidence that in functionally distinct NP cell types, EGR1 mediates specialized cellular responses to either growth factor stimulation or inflammatory cytokines. The higher induction of EGR1 in NP-R cells may reflect an intrinsic ability to respond more robustly to differentiation conditions than a NP-nR clone [6]. The ability of cells to generate a physiologically appropriate response is dictated by its epigenetic constitution: as a result of lineage commitment and differentiation cells have become epigenetically restricted to function within a given lineage. Although IEGs play crucial roles in helping cells respond to, for instance, differentiation factors, increasing experimental evidence suggests that their main task is to augment rather than initiate gene transcriptional activity [37, 38]. This ability is at least in part due to their recruitment of Histone Acetyl Transferase (HAT) activity to their transcriptional target sites [39]. Likewise we found that early global histone acetylation failed in EGR1-deficient chondrogenic cells [16]. Moreover, we showed that, despite the presence of consensus EGR1 binding sites, Polycomb-dependent, epigenetically repressive Histone H3 lysine 27 trimethyl (H3K27me3) marking prevented promoter access of EGR1 [16]. We propose that restrictive epigenetic boundaries dictate the cell-specific transcriptomic responses in NP-R cells (more anabolic) versus NP-nR cells (more catabolic). In differentiating ATDC5 cells we were able to show that expression of certain lineage-specific genes (*SOX6, AGC*) was attained through loss of repressive H3K27me3 marks [16]. It is conceivable that similar epigenetic programming has occurred during maturation of NP-R to NP-nR cells. Although VPA was merely used as a positive response control in the current study, it is interesting to note that NP-R and NP-nR cells also responded differentially to VPA. VPA is known to suppress IEG responses [24, 25]. It is conceivable that the observed differences relate to a relative insensitivity of NP-R cells to VPA (*i.e.* epigenotype). Involvement of restricted epigenomic vulnerability to VPA may be corroborated by studies that show VPA has teratogenic effects in certain species [40, 41] and during defined time windows during embryogenesis [42].

Proteins of the matrix metalloproteinase (MMP) family are involved in the breakdown of extracellular matrix in normal physiological processes, such as embryonic development, reproduction, and tissue remodeling, as well as in disease processes, such as arthritis and metastasis [43–45]. EGR1 was found to mediate TNFα-induced *MMP9* expression in various cell types [18]. Our data are in line with an early role for TNFα in the catabolic response of NP-nR cells [28], as it is rapidly and exclusively induced by IL-1β in NP-nR cells. Interestingly, EGR1 appears to restrain *TNFα* expression rather than to augment it. Indeed, EGR1 is known to positively and negatively affect gene expression, through partnerships with transcriptional co-activators or co-repressors [26, 31–33].

Interestingly, both NP cell types do not induce *COX2* mRNA in response to IL-1β; EGR1 clearly functions to suppress *COX2* expression, as both NP-nR and NP-R clones show strong *COX2* induction at 48 h of IL-1β treatment in the absence of EGR1 (60,000 and 40,000 fold, respectively). We previously reported that *COX2* expression correlates with hypertrophic differentiation in articular cartilage models and pharmacological inhibition of COX2 decreases hypertrophy [29]. It is conceivable that *COX2* induction, as part of a first line response, needs to be avoided to prevent (irreversible) DDD. *COX2* expression was specifically observed in degenerate discs [46] and a correlation between DDD and cellular hypertrophy in the NP has been observed before [30]. In line with this thought: EGR1 was shown to be involved in repair of mesenchymal tissues [14, 15, 47]. It is of considerable interest to establish whether and when a hypertrophic response is initiated by these NP-nR cells and whether this is connected to loss of EGR1. A similar rational may be envisioned pertaining to keeping *TNFα* induction in check: this would prevent full blown release of COX2, MMP13, ADAMTS5 and Syndecan4 [48]. Despite the observed effects of EGR1 knock-down on transcriptional responses in NP cells, this data does not provide evidence for direct promoter binding of EGR1. Chromatin-immunoprecipitation analysis of EGR1-binding and specific histone modifications at these loci, as a function of response time, would shed additional light on their exact role in respect to the observed transcriptional response profiles.

### Relevance EGR1 signaling in vivo

Classical genetic knock-out mice for *EGR1* show no overt phenotype associated with bone and/or cartilage development, likely due to activation of functionally redundant mechanisms (e.g. EGR2-4) in vivo that compensate for loss of EGR1 function during development. When models of acute plasticity are interrogated however, EGR1 function becomes clear in vivo and in vitro. Following these strategies, it was established that EGR1 is important for adipogenesis and tenogenesis [13–15]. EGR1 (NGFI1, KROX24) and family members also mediate gene-environment interaction in the brain and is responsible for *i.a.* dendritic spine formation and synaptic plasticity and controls higher brain functions including movement, emotions, learning and memory [49–53]. Of relevance to the current study, EGR1 and EGR2 have been associated with inflammatory responses at multiple levels [17–20, 54]. We find that EGR1 is responsible for anabolic and catabolic responses of distinct NP cell types. EGR1 was shown to inhibit ECM molecule production in chondrocytes exposed to TNFα [27]. We and others have also shown that EGR1 is required for tissue development and repair [16, 21, 22]. Combined, these observations stress the importance of IEGs in general and of EGR1 in particular in plastic biological systems. Identification of downstream signaling components within cell type-specific EGR1-dependent response pathways is expected to contribute to developing therapeutic strategies for e.g. degenerative disc disease.

## Conclusion

EGR1 functions at the cross roads of inflammation and repair and represents an important response factor that allows NP cells to adapt to anabolic or catabolic stimuli. Well-defined cell models like the ones described herein will be imperative to study the role of specific cellular subtypes in degenerative disc disease and their potential use in cell-based disc regeneration strategies.

## Additional file

Additional file 1: Figure S1. Optimization of EGR1 knock-down using siRNA, A) cells were transfected with the indicated concentrations of siRNA against *EGR1* mRNA (3, 30 and 100 nM). 30 nM and 100 nM showed significant (*; *p* < 0.05) reduction of *EGR1* expression compared to *siCTRL*. B) Absence of off-target effects in *siEGR1* treated cells. *ERG1*, *EGR2* and *EGR3* mRNA levels are depicted in *siCTRL*- and in *siEGR1*-treated cells. Only *EGR1* mRNA levels were significantly reduced (*; *p* < 0.05). C) Sustained *EGR1* knock-down: NP-nR (nR) and NP-R (R) cells were stimulated with ITS medium in the presence of control (*CTRL*; 30 nM) or *EGR1* siRNA (*EGR1*; 30 nM); EGR1 protein expression was measured at 0, 2 and 48 hours post- stimulation in both cell lines. Βeta Actin (βACT) was used as loading control; the indication (t+) points to a relatively long apposition time. (TIF 59 kb)

## Abbreviations

ADAMTS4: a disintegrin-like and metalloproteinase with thrombospondin type 1 motif 4
AF: annulus fibrosus
COL1A1: collagen type I, α1
COL2A1: collagen type II, α1
COX2: cyclooxygenase 2
CYCLO: cyclophillin B
DDD: degenerative disc disease
ECM: extra cellular matrix
EGR1: early growth response factor 1
EIF2B1: eukaryotic translation initiation factor 2B, subunit 1
IEG: immediate early gene
IL-1β: interleukin 1 beta
IVD: intervertebral disc
KRT19: keratin 19
MMP3: matrix metalloproteinase 3
MRPL19: mitochondrial ribosomal protein L19
NC: notochordal cell
NFκB: nuclear Factor kappa B
NP: nucleus pulposus
NP-(n)R: NP-(non)responder cells
siCTRL: scrambled siRNA (control)
siEGR1: siRNA against EGR1 mRNA
SOX9: SRY-box 9
T: brachyury T
TNFα: tumor necrosis factor alpha
VCAN: versican
VPA: valproic acid
βACT: beta actin
