# Supplementary figures and images for: EGR1 controls divergent cellular responses of distinctive nucleus pulposus cell types
Source: BMC Musculoskelet Disord. 2016 Mar 14;17:124. doi: 10.1186/s12891-016-0979-x (PMC4791893; doi:10.1186/s12891-016-0979-x)

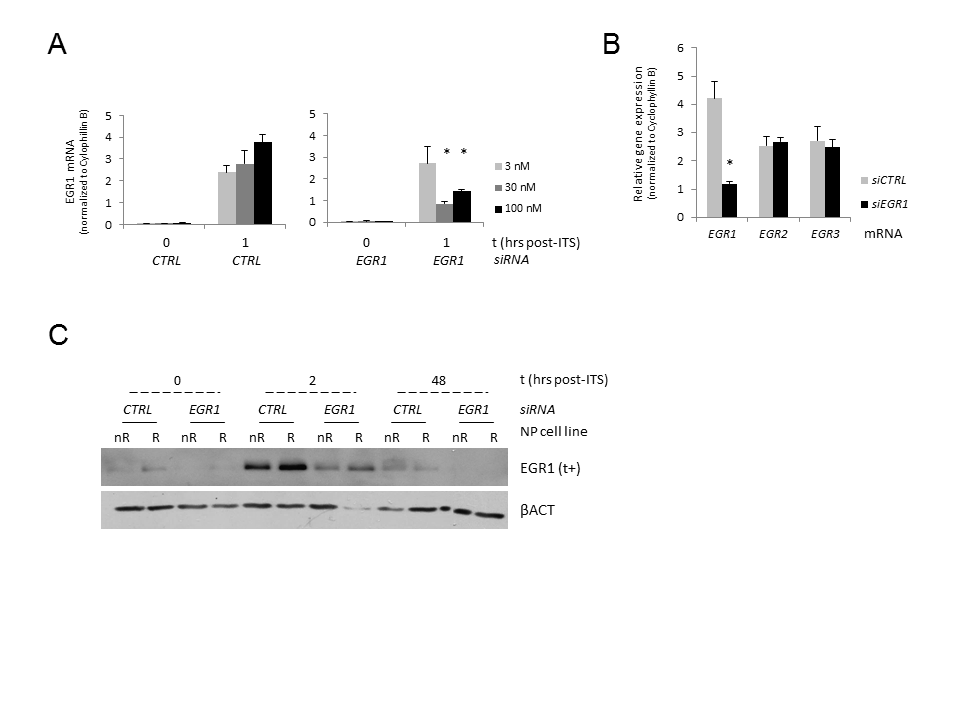

Supplement: Additional file 1: Figure S1. — Optimization of EGR1 knock-down using siRNA, A) cells were transfected with the indicated concentrations of siRNA against EGR1 mRNA (3, 30 and 100 nM). 30 nM and 100 nM showed significant (*; p < 0.05) reduction of EGR1 expression compared to siCTRL. B) Absence of off-target effects in siEGR1 treated cells. ERG1, EGR2 and EGR3 mRNA levels are depicted in siCTRL- and in siEGR1-treated cells. Only EGR1 mRNA levels were significantly reduced (*; p < 0.05). C) Sustained EGR1 knock-down: NP-nR (nR) and NP-R (R) cells were stimulated with ITS medium in the presence of control (CTRL; 30 nM) or EGR1 siRNA (EGR1; 30 nM); EGR1 protein expression was measured at 0, 2 and 48 hours post- stimulation in both cell lines. Βeta Actin (βACT) was used as loading control; the indication (t+) points to a relatively long apposition time. (TIF 59 kb) [file 12891_2016_979_MOESM1_ESM.tif]
